# Diagnostic reassessment in myeloproliferative neoplasms: the value of functional iron parameters and JAK2 allelic burden

**DOI:** 10.1007/s00277-026-06774-y

**Published:** 2026-01-24

**Authors:** Rita González-Resina, Sara España-Fernández de Valderrama, Ángeles Montañés, Valle Recasens, Gonzalo Caballero

**Affiliations:** 1 Servicio de Hematología y Hemoterapia, Hospital Universitario Miguel Servet, Zaragoza, Spain; 2 Servicio de Cirugía Ortopédica y Traumatología, Hospital Universitario Miguel Servet, Zaragoza, Spain; 3 Departamento de Medicina, Psiquiatría y Dermatología, Facultad de Medicina, Universidad de Zaragoza, Zaragoza, Spain

**Keywords:** Polycythemia vera, Essential thrombocythemia, Transferrin saturation index, *JAK2* variant allele frequency, Erythropoietin, Iron metabolism

## Abstract

Polycythemia vera (PV) and essential thrombocythemia (ET) are chronic myeloproliferative neoplasms (MPNs), often associated with mutations in *JAK2*, *CALR*, and *MPL*. Differentiating PV from ET can be challenging in borderline cases, particularly when hemoglobin (Hb), hematocrit (Hct) and erythropoietin (EPO) values are inconclusive. Functional iron parameters and *JAK2* variant allele frequency (VAF) may provide additional discriminatory value. To assess the diagnostic utility of transferrin saturation index (TSI), serum ferritin, EPO, and *JAK2* VAF in distinguishing PV from ET, and to evaluate their association with mutational profiles. We conducted a retrospective, single-center study including 260 adult patients diagnosed with PV or ET between 2009 and 2024. Demographic, clinical, molecular, and laboratory parameters—including ferritin, TSI, EPO, Hb, Hct, and *JAK2* VAF—were analyzed. Comparative and correlation analyses were performed using appropriate statistical tests. Compared to ET, patients with PV had significantly lower ferritin (median: 35.65 vs. 95.05 ng/mL), TSI (12.9% vs. 21.64%), and EPO (2.23 vs. 6.11 mIU/mL), but higher Hb (17.7 vs. 14.3 g/dL) and Hct (54.6% vs. 43.0%) (all p < 0.001). TSI discriminated PV from ET better than ferritin (p < 0.001 vs. p = 0.128). Among JAK2-mutated cases, VAF was higher in PV than ET (median: 48% vs. 21%, p = 0.003). VAF correlated inversely with ferritin, TSI, and EPO, and positively with Hct. TSI and *JAK2* VAF outperform ferritin as diagnostic markers to differentiate PV from ET. Integrating functional iron parameters with molecular data improves diagnostic accuracy, particularly in clinically ambiguous cases, and supports their inclusion in MPN diagnostic algorithms.

## Introduction

Chronic myeloproliferative neoplasms (CMPNs) are a heterogeneous group of hematological disorders characterized by clonal proliferation of one or more myeloid cell lines in the bone marrow. These diseases are frequently associated with mutations in the *JAK2* genes, including *JAK2* V617F and *JAK2* exon 12 [1, 2], as well as *CALR* and *MPL*, which impact their pathophysiology and clinical course. Among CMPNs, polycythemia vera (PV) and essential thrombocythemia (ET) present distinctive alterations in iron metabolism and erythropoietin (EPO) levels, reflecting the pathophysiological characteristics of each disease and their impact on erythropoiesis [3, 4].

In PV, the overproduction of erythrocytes leads to functional iron deficiency, attributed to the depletion of iron stores by increased erythropoiesis and therapeutic phlebotomies [5, 6]. Consequently, the majority of patients exhibit diminished serum ferritin levels and a reduced transferrin saturation index (TSI) from the time of diagnosis onwards. At the molecular level, an abnormality in the regulation of hepcidin, a hormone pivotal to iron homeostasis, has been identified. Serum levels of hepcidin may be reduced in patients with PV, which promotes greater intestinal iron absorption and alters its availability for erythropoiesis [3]. Furthermore, EPO levels in PV are frequently disproportionately low in relation to the degree of erythrocytosis, in contrast to other causes of polycythemia, where EPO levels are elevated.

In contrast, although the iron profile in ET is not as clearly characterized as in PV, some studies suggest that patients may have normal or slightly decreased ferritin levels. However, the availability of iron has been observed to modulate the disease phenotype, as evidenced by studies utilizing mouse models with *JAK2* mutations [4]. Levels of EPO in ET are typically normal or slightly elevated, contingent on the presence of concomitant anemia or other conditions that influence its production.

An understanding of the characteristics of the iron profile and EPO levels has become increasingly important due to their relevance in the differential diagnosis of CMPNs. The influence of these parameters on hemoglobin (Hb) and hematocrit (Hct) levels can impede the accurate classification of patients with histological characteristics indicative of PV, but whose Hb or Hct values do not attain the stipulated diagnostic thresholds.

The objective of this study is to make a comparison between the iron, erythropoietic and mutational profiles of patients with ET and PV, with particular attention to the diagnostic value of IST versus ferritin, as well as the usefulness of the *JAK2* allele variant fraction (VAF) in discriminating between the two entities.

## Materials and methods

A single-centre, retrospective observational analysis was conducted, including 260 adult patients diagnosed with primary PV or essential ET type myeloproliferative neoplasm (MPN), according to the World Health Organization (WHO) criteria in force at the time of diagnosis and prior to the initiation of any therapy. Patients were diagnosed between May 2009 and December 2024, and inclusion was performed consecutively from institutional clinical records. Exclusion criteria were incomplete baseline data for the primary analyses, including missing *JAK2* VAF or iron parameters.

### Mutations study

The mass sequencing study was conducted at the Molecular Diagnosis Unit of the Haematology Service of the Cancer Research Centre in Salamanca. The Healthincode-Haematology OncoKitDx (Genycell) panel was utilised on the MiSeqDx (Illumina) instrument. The bioinformatic analysis was performed using Data Genomics software.

### Statistical analysis

The demographic (age, sex), clinical (diagnosis of PV or ET) and molecular variables were collected for each patient at the time of diagnosis, including the presence of mutations in the *JAK2* (V617F or exon 12), *CALR* (type 1, type 2 or other variants) and *MPL* (W515L, S505N) genes. Patients without identified mutations in these genes were classified as triple negative. The analytical parameters evaluated included serum ferritin concentration (ng/mL), IST (%), EPO (mIU/mL), Hb (g/dL), Hct (%), and, in the case of *JAK2*-mutated patients, VAF (%).

The distribution of quantitative variables was assessed using the Shapiro-Wilk test. As the data did not conform to a normal distribution, the results were expressed as median and interquartile range (IQR) and then compared between groups using the non-parametric Mann-Whitney U test and the Kruskal-Wallis test. Categorical variables were expressed as absolute frequencies and percentages and were compared using the chi-square test or Fisher’s exact test, as appropriate. Pearson’s correlation coefficient was utilized to investigate the association between *JAK2* allele load (VAF) and biochemical parameters. In a similar manner, a binary logistic regression analysis was conducted in order to evaluate the capacity of iron parameters (ferritin, IST) to differentiate between primary vitreous (PV) and traction eye (TE). All statistical analyses were performed with a two-tailed significance threshold set at *p* < 0.05. The statistical analysis was conducted using SPSS^®^ software (version 25.1).

## Results

### General characteristics of the cohort

A total of 260 patients diagnosed with CMPNs were included in the study, of whom 59.6% (*n* = 155) were diagnosed with TE and 40.4% (*n* = 105) with PV. The cohort comprised 58.9% (*n* = 153) women and 41.1% (*n* = 107) men. The median age at diagnosis was 69 years, with an IQR of 22 years. From a molecular perspective, the *JAK2* gene mutation was the most prevalent, observed in 74.2% of patients (*n* = 193), with a marked predominance of the V617F variant (99.5%) and a single case with a mutation in exon 12 (0.5%). Mutations in the *CALR* gene were detected in 12.7% of cases (*n* = 33), with the type 1 mutation (52 bp deletion, del52) being the most prevalent (66.7%). Mutations in the *MPL* gene were observed in 4.2% of patients (*n* = 11), with the W515L variant predominating (72.7%). In the remaining 8.8% of cases, no mutations were identified in any of these three genes. The baseline clinical characteristics and mutational distribution of the cohort of patients with CMPNs are shown in Table 1.

**Table 1:** Clinical characteristics and mutation frequencies in patients with CMPNs.* CMPNs* chronic myeloproliferative neoplasm, *ET* essential thrombocythemia, *PV* polycythemia vera, *IQR* interquartile range

	CMPNs (n=260)
Sex, n (%)	
Male	107 (41,1)
Female	153 (58,9)
Age, median (IQR)	69 (22)
Type of CMPNs, n (%)	
TE	155 (59,6)
PV	105 (40,4)
Mutations, n (%)	
* JAK2*	**193 (74,2)**
V617F	192 (99,5)
Exon 12	1 (0,5)
* CALR*	**33 (12,7)**
Type 1	22 (66,7)
Type 2	8 (24,2)
Del30bp	1 (3,0)
Unknown	2 (6,1)
* MPL*	**11 (4,2)**
W515L	8 (72,7)
S505N	2 (18,2)
W515K	1 (9,1)
Negative	**23 (8,8)**

## Clinical and biochemical comparison between PV and TE

Patients diagnosed with PV were found to be older at the time of diagnosis (median: 71 vs. 68 years; *p* = 0.006). In relation to gender, a higher proportion of women was observed in the ET group (64.5% vs. 50.5%; *p* = 0.024). In the biochemical analysis, patients with PV exhibited significantly lower ferritin levels (35.65 ng/mL; reference interval [RI]: 65.7) compared to those with TE (95.05 ng/mL; RI: 93.1) (*p* < 0.001). Similarly, IST values (12.9% vs. 21.64%; *p* < 0.001) and EPO values (2.23 vs. 6.11 mIU/mL; *p* < 0.001) were found to be lower in patients with PV. Conversely, the levels of Hb (17.65 vs. 14.3 g/dL) and Hct (54.6% vs. 43.0%) were found to be significantly higher in patients with PV (*p* < 0.001 in both cases).

From a molecular point of view, significant differences were observed in the mutational profile between patients diagnosed with PV and ET (*p* < 0.001). The *JAK2* mutation was observed to be more prevalent in patients diagnosed with PV (93.3%) compared to those diagnosed with ET (61.3%). Conversely, *CALR* and *MPL* mutations were exclusively detected in patients with ET. In a similar vein, the ET group exhibited a higher percentage of cases devoid of identifiable mutations (10.3% vs. 6.7%). The distribution of mutations according to diagnosis is shown in Table 2.

A logistic regression study was conducted to evaluate the diagnostic discriminatory capacity of the iron profile between both CMPNs. TSI was found to be a significant predictor of the diagnosis of PV versus TE (*p* = 0.000), while ferritin did not reach statistical significance (*p* = 0.128). This suggests that TSI may have greater utility as a marker of iron deficiency in this context.

**Table 2:** Clinical, molecular, and analytical comparison between patients with ET and PV. ET: essential thrombocythemia. *PV* polycythemia vera, *IQR* interquartile range, *TSI* transferrin saturation index, *EPO* erythropoietin, *hb* hemoglobin, *hct* hematocrit

	ET (*n *= 155)	PV (*n *= 105)	*p*-value
Age, median (IQR)	68,00 (20)	71,23 (23)	0,006
Sex, n (%)			0,024
Male	55 (33,5)	52 (49,5)	
Female	100 (64,5)	53 (50,5)	
Mutations, n (%)			0,000
* JAK2*	95 (74,2)	98 (93,3)	
* CALR*	33 (21,3)	NA	
* MPL*	11 (7,1)	NA	
Negative	16 (10,3)	7 (6,7)	
Variable, median (IQR)			
Ferritine (ng/mL)	95,05 (93,1)	35,65 (65,7)	0,000
TSI (%)	21,64 (11,13)	12,9 (16,07)	0,000
EPO (mUI/mL)	6,11 (5,06)	2,23 (1,6)	0,000
Hb (g/dL)	14,3 (1,8)	17,65 (3,52)	0,000
Hct (%)	43,0 (5,65)	54,6 (9,78)	0,000

### Analytical analysis according to mutational profile

Patients diagnosed with CMPN and *JAK2* mutations exhibited significantly lower levels of ferritin (51.8 ng/mL; IQR: 80.4), TSI (16.79%; IQR: 15.45), and EPO (3.00 mIU/mL; RI: 3.41) in comparison to those with *CALR* or *MPL* mutations or no identifiable mutations, exhibiting statistically significant differences (*p* = 0.006, *p* = 0.002, and *p* < 0.001, respectively). Furthermore, a divergence in age was identified, with patients exhibiting *JAK2* and *MPL* mutations showing higher age averages (*p* = 0.003). Additionally, significant variations were observed in Hb and Hct levels among the molecular subgroups (*p* < 0.001 in both instances) (Table 3).

**Table 3:** Analytical parameters according to mutation profile. IQR: interquartile range. TSI: transferrin saturation index. EPO: erythropoietin. Hb: hemoglobin. Hct: hematocrit

Variable, median (IQR)	JAK2 (*n* = 193)	CALR (*n* = 33)	MPL (*n* = 11)	Negative (*n* = 23)	*p*-value
Ferritine (ng/mL)	51,8 (80,4)	118,4 (60,2)	113,2 (91,1)	90,90 (65,3)	0,006
TSI (%)	16,79 (15,45)	18,46 (10,47)	24,35 (10,39)	25,44 (8,86)	0,002
EPO (mUI/mL)	3,00 (3,41)	10,68 (3,66)	8,71 (5,79)	6,13 (8,25)	0,000
Hb (g/dL)	15,3 (3,6)	12,8 (2,3)	14,1 (3,5)	16,0 (5,2)	0,000
Hct (%)	46,7 (11,5)	39,7 (6,7)	42,5 (9,4)	46,7 (14,8)	0,000

### Analytical subanalysis in patients with *JAK2*-positive CMPN

In the subgroup of CMPN with *JAK2* mutation, the results of the iron profile and VAF were compared in patients diagnosed with ET and PV. No statistically significant differences were identified in ferritin (82.1 vs. 34.0 ng/mL; *p* = 0.356), TSI (21.56% vs. 12.18%, *p* = 0.345) or EPO (4.64 vs. 2.12 mIU/mL; *p* = 0.134). However, VAF was found to be significantly higher in PV (48%; CI: 34) than in ET (21%; CI: 23) (*p* = 0.003), thereby reinforcing its usefulness as a discriminatory marker between the two entities (see Table 4 for details).

**Table 4:** Comparison of analytical parameters and VAF between patients with *JAK2*-mutated CMPN according to diagnosis. VAF: variant allele fraction, IQR: interquartile range. TSI: transferrin saturation index, EPO: erythropoietin, hb: hemoglobin, hct: hematocrit

Variable, median (IQR)	ET JAK2 (*n* = 95)	PV JAK2 (*n* = 98)	*p*-value
Ferritine (ng/mL)	82,1 (97,8)	34,0 (61,1)	0,356
TSI (%)	21,56 (13,78)	12,18 (15,62)	0,345
EPO (mUI/mL)	4,64 (3,55)	2,12 (1,38)	0,134
VAF (%)	21,0 (23)	48,0 (34)	0,003

## Association between VAF and analytical parameters

A significant negative correlation was observed between VAF and TSI (*r* = − 0.420; *p* < 0.001), as well as with serum ferritin (*r* = − 0.216; *p* = 0.003) and EPO levels (*r* = − 0.277; *p* < 0.001). These findings suggest that a higher mutational burden is associated with greater functional iron deficiency and lower endogenous erythropoietic stimulation. In contrast, no significant correlation was found between VAF and Hb levels (*r* = 0.054; *p* = 0.456). Conversely, a substantial positive correlation was identified with Hct (*r* = 0.439; *p* < 0.001), suggesting that an elevated mutational burden is associated with an increased concentration of circulating erythrocytes.

### Comparative analysis in patients with essential thrombocythemia according to *JAK2* mutation status

Within the cohort of patients diagnosed with ET, biochemical parameters were compared between those with a *JAK2* mutation and those with other mutations or who were triple negative. Patients with *JAK2*-mutated ET exhibited significantly lower ferritin levels (81.9 ng/mL; IQR: 93.33) in comparison to the control group (113.1 ng/mL; IQR: 79.85; *p* = 0.003). Additionally, they showed a lower TSI (21.6% vs. 22.84%; *p* = 0.006) and lower EPO levels (4.67 vs. 9.12 mIU/mL; *p* = 0.036). In contrast, Hb (14.45 vs. 13.85 g/dL; *p* < 0.001) and Hct (43.5% vs. 41.2%; *p* = 0.008) levels were higher in patients with *JAK2* mutation (Table 5). These findings serve to reinforce the notion that mutational status exerts a significant influence on the manifestation of the hematological phenotype, even within the context of the same clinical entity.

**Table 5:** Analytical parameters in patients with ET according to *JAK2* mutation status. IQR: interquartile range, TSI: transferrin saturation index, EPO: erythropoietin, hb: hemoglobin, hct: hematocrit

Variable, median (IQR)	ET JAK2 (*n* = 95)	no-JAK2 ET (*n* = 60)	*p*-value
**Ferritine** (ng/mL)	81,9 (93,33)	113,10 (79,85)	0,003
**TSI** (%)	21,6 (13,69)	22,84 (9,17)	0,006
**EPO** (mUI/mL)	4,67 (3,76)	9,12 (5,24)	0,036
**Hb** (g/dL)	14,45 (1,60)	13,85 (2,25)	0,000
**Hct** (%)	43,5 (4,25)	41,2 (8,48)	0,008

## Discussion

The present retrospective study compared the iron-related, erythropoietic, and mulecular parameters in patients with PV and ET, with the purpose of identifying variables that may assist in the differential diagnosis between these two CMPNs. As expected from prior literature, patients with PV showed lower ferritin, TSI, and EPO levels together with higher Hb and Hct values, consistent with a phenotype of expanded erythropoiesis in the setting of functional iron deficiency [7, 8]. Altered regulation of the hepcidin-iron axis has been implicated in this process [3, 9], and our finding align with those reports. Conversely, patients with ET generally exhibited iron parameters within or near the normal range, which parallels previous observations in this disease [10, 11].

An interesting observation from our analysis was that TSI displayed a trend toward greater discriminatory value than ferritin when distinguishing PV from ET. This pattern, suggested by logistic regression analysis, could be partly explained by the well-known susceptibility of ferritin to inflammation and comorbid conditions [12]. In this context, TSI may offer a more direct reflection of functional iron availability. Nevertheless, this interpretation requires confirmation in prospective studies with standardized assessment of inflammatory markers and iron supplementation.

From a molecular standpoint, our cohort showed distributions broadly consistent with previously reported MPN series: *JAK2* V617F mutations were highly prevalent in PV and present in a substantial proportion of ET vases, whereas *CALR* and *MPL* mutations were restricted to ET [13, 14]. *JAK2* VAF values were higher in PV than in ET, in line with prior reports suggesting that the allelic burden may contribute to phenotypic variation across the MPN spectrum [15, 16]. We also observed inverse correlations between *JAK2* VAF and ferritin, TSI and EPO levels, as well as a positive correlation with Hct. These associations may reflect the relationship between higher mutational burden and a more proliferative erythroid phenotype, although causality cannot be inferred from these data. Similar trends have been described in previous studies linking higher VAF with increased erythrocyte mass and thrombotic risk [17, 18].

Within the ET group, patients carrying *JAK2* mutations exhibited iron-related and erythropoietic features that appeared closer to those typically seen in PV, including lower ferritin, TSI and EPO levels and higher Hb and Hct. These observations are compatible with the notion that mutational status and allelic burden modulate phenotypic expression within ET [18].

This study has several limitations that must be considered. It was conducted in a single center and included a moderately sized cohort, which may limit the generalizability of the findings. Although all biological parameters were collected only at the time of diagnosis, potential confounders with an impact on iron metabolism – such as prior iron supplementation, underlying inflammatory conditions or subsequent therapeutics approaches, including interferon or ruxolitinib, which are known to have a potential impact on *JAK2* VAF – were not systematically recorded and therefore could not be assessed. These observations underscore the need for longitudinal reassessment throughout the course of the disease, as well as validation through prospective multicenter studies with standardized data collection.

In summary, the integration of iron-related parameters and molecular data at diagnosis may offer complementary insights when distinguishing PV from ET, particularly in cases with borderline clinical or analytical features. However, the extent to which these markers improve diagnostic precision remains to be clarified in future prospective studies.

## Conclussions

In this single-center retrospective cohort, TSI, EPO and *JAK2* VAF showed potential to provide complementary diagnostic information when differentiating PV from ET at the time of diagnosis. Patients with ET carrying *JAK2* mutations exhibited features partially overlapping with those of PV, suggesting a modulatory role of mutational status and allelic burden in phenotypic expression. These finding should be considered exploratory and require validation in prospective multicenter studies with standardized assessment of iron metabolism and molecular parameters.

## Data Availability

The datasets analyzed during the current study derive from the GEMFIN registry and from hospital clinical records. Due to patient privacy and institutional restrictions, these data are not publicly available. De-identified data may be obtained from the corresponding author on reasonable request and subject to GEMFIN approval.
